# Tyro3, Axl, and Mertk receptors differentially participate in platelet activation and thrombus formation

**DOI:** 10.1186/s12964-018-0308-0

**Published:** 2018-12-12

**Authors:** Junsong Zhou, Aizhen Yang, Yucan Wang, Fengwu Chen, Zhenzhen Zhao, Viralkumar Davra, Katsue Suzuki-Inoue, Yukio Ozaki, Raymond B. Birge, Qingxian Lu, Yi Wu

**Affiliations:** 1 Cyrus Tang Hematology Center, Collaborative Innovation Center of Hematology, State Key Laboratory of Radiation Medicine and Protection, Soochow University, Suzhou, 215123 China; 2 Department of Microbiology, Biochemistry and Molecular Genetics, Rutgers University-New Jersey Medical School, Newark, NJ USA; 3 Department of Clinical and Laboratory Medicine, Faculty of Medicine, University of Yamanashi, 1110 Shimokato, Chuo, Yamanashi, 409-3898 Japan; 4 Department of Ophthalmology and Visual Sciences, University of Louisville, Louisville, KY 40202 USA; 5 The Sol Sherry Thrombosis Research Center, Temple University School of Medicine, 3420 North Broad Street, Philadelphia, PA19140 USA

**Keywords:** Gas6 receptors, Platelet, Activation, Thrombosis, Trans interaction

## Abstract

**Background:**

Previously, several studies have shown that Tyro3, Axl, and Mertk (TAM) receptors participate in platelet activation and thrombosis. However, the role of individual receptors is not fully understood.

**Methods:**

Using single receptor-deficient platelets from TAM knockout mice in the C57BL/6 J strain, we performed a knockout study using single TAM-deficient mice. We treated platelets isolated from TAM knockout mice with the Glycoprotein VI (GPVI) agonists convulxin, poly(PHG), and collagen-related triple-helical peptide (CRP), as well as thrombin for in-vitro experiments. We used a laser-induced cremaster arterial injury model for thrombosis experiments in vivo.

**Results:**

Deficiency of the tyrosine kinase receptors, Axl or Tyro3, but not Mertk, inhibited aggregation, spreading, JON/A binding, and P-selectin expression of platelets in vitro. In vivo, platelet thrombus formation was significantly decreased in Axl^−/−^ and Tyro3^−/−^ mice, but not in Mertk^−/−^ mice. Upon stimulation with glycoprotein VI (GPVI) agonists, tyrosine phosphorylation of signaling molecules, including spleen tyrosine kinase (Syk) and phospholipase C-γ2 (PLCγ2), was decreased in Axl^−/−^ and Tyro3^−/−^ platelets, but not in Mertk^−/−^ platelets. While platelet aggregation induced by agonists did not differ in the presence or absence of the Gas6 neutralizing antibody, the platelet aggregation was inhibited by anti-Axl or anti-Tyro3 neutralizing antibodies antibody, but not the anti-Mertk antibody. Additionally, the recombinant extracellular domain of Axl or Tyro3, but not that of Mertk, also inhibited platelet aggregation.

**Conclusions:**

These data suggest that Axl and Tyro3, but not Mertk, have an important role in platelet activation and thrombus formation, and mechanistically may do so by a pathway that regulates inside to outside signaling and heterotypic interactions via the extracellular domains of TAMs.

**Electronic supplementary material:**

The online version of this article (10.1186/s12964-018-0308-0) contains supplementary material, which is available to authorized users.

## Backgroud

Tyro3, Axl, and Mertk comprise the members of the TAM family of receptor tyrosine kinases that participate in a number of important physiological functions that include the clearance of apoptotic cells, resolution of inflammation, as well as platelet aggregation and clot formation. Structurally, TAM receptors share a characteristic domain organization comprising two extracellular immunoglobulin-like domains, two fibronectin type III-like domains, and a C-terminal cytoplasmic tyrosine kinase domain [1]. For conventional outside-to-inside signaling, the main endogenous ligands, Gas6 and Protein S, bind to the TAM receptors via its sex hormone-binding globulin-like domain and activate the intracelluar kinase domain [2–8]. Notably, It has been observed that Gas6 binding to TAM receptors have multiple cell intrinsic roles that regulate cell growth [9], cell survival [10–12], apoptosis [13–15], cell proliferation [10, 16, 17], cell adhesion [6, 18–21], and in professional phagocytes apoptotic cell clearance (efferocytosis) [22].

Previously, several studies have shown that TAM receptors (and Gas6) also participate in platelet activation and thrombosis [23–27]. However, the conclusions regarding the function of each TAM receptor is not fully understood. For example, Angelillo-Scherrer et al have shown that deficiency of Gas6 and TAM receptors causes platelet dysfunction and protects mice against thrombosis, and that TAM receptors are equally important in platelet activation [23, 24]. In contrast, other studies using antibodies proposed that the TAM receptors play a distinct role in platelet function [28] and are selectively involved in ADP-mediated platelet activation, but other receptors, such as collagen receptors, are not involved [27]. Furthermore, Chen et al. showed that platelets only express Mertk but not Tyro3 and Axl, and the recombinant extracellular domain of Mertk inhibited platelet aggregation induced by collagen [26]. Clearly, the role of TAM receptors in platelet activation still remains inconclusive and issues need to be addressed such as whether Gas6 receptors are required for platelet activation and if Axl, Tyro3, and Mertk play an equal role in platelet activation and thrombosis. It is important to address these issues because it will not only help understand the specific functions of these TAM receptors in platelet activation and thrombosis, but it may also reveal the potential target for anti-thrombosis treatment.

In this study, we tested single TAM-knockout and found that the deficiency of Axl or Tyro3 markedly inhibited platelet aggregation, integrin αIIbβ3 activation, granule secretion, platelet spreading, intracellular tyrosine phosphorylation, and platelet thrombus formation in vivo. Correspondingly, blockade of Axl or Tyro3 by specific antibodies and recombinant extracellular domains suppressed platelet activation. In contrast, neither the deficiency of Mertk nor the inhibition of Mertk affected platelet activation. These observations provide evidence that Axl and Tyro3 play an important role in platelet activation and thrombosis, and may serve as a better target than Mertk for inhibition of thrombosis.

## Methods and materials

### Mice

The knockout mice with deficiency of Mertk, Axl and Tyro-3 were generated as previously described [29].

### Preparation of washed platelets

All studies on human platelets were performed after approval by the Institution Review Board. Platelets were prepared as described previously [30, 31]. Briefly, 8.6 mL of blood was drawn into 1.4 mL of acid-citrate dextrose (ACD) solution (65 mM sodium citrate, 70 mM citric acid, and 100 mM dextrose, pH 4.4). After centrifugation at 250 x g for 20 min, platelet-rich plasma (PRP) was collected and gel-filtered on a Sepharose 2B column equilibrated in a Tyrode-albumin solution. Mouse blood was drawn by inferior vena cava puncture after anesthesia by pentobarbital (50 mg kg^− 1^) [30]. Blood was collected into ACD and was diluted (1:3) with modified Tyrode’s buffer (137 mM NaCl, 20 mM HEPES, 5.6 mM glucose, 1 g L^− 1^ BSA, 1 mM MgCl_2_, 2.7 mM KCl, and 3.3 mM NaH_2_PO4, pH 7.4). Blood was centrifuged at 230 g for 10 min, to obtain platelet-rich plasma (PRP). To prepare washed platelets, PEG1 (final concentration, 1 μM) and apyrase (final concentration, 0.2 U mL^− 1^) was added to PRP. Washed platelets were prepared by centrifuging PRP at 980 g for 15 min and platelet pellets were resuspended in modified Tyrode’s buffer.

### Platelet aggregation

Platelet aggregation was monitored by measuring light transmission with the use of an aggregation analyzer (CHRONO-LOG Corporation, 560CA). The instrument was calibrated with a washed platelet suspension (3 × 10^8^ platelets mL^− 1^) for zero light transmission and with buffer for 100% transmission. Aggregation was initiated by addition of agonists under constant stirring at 1200 rpm at 37 °C. CaCl_2_ were added at final concentration of 1 mM immediately before platelet stimulation.

### Platelet spreading assay

Coverslips were coated with 10 μg mL^− 1^ fibrinogen overnight at 4 °C and blocked with 1% fatty acid-free BSA. Washed platelets in Tyrode’s buffer supplemented with 1 mM CaCl_2_ and agonist were seeded on coverslips and incubated at 37 °C for 30 min. After washing and fixation with 4% paraformaldehyde, the adherent platelets were stained with TRITC-conjugated phalloidin (F-actin stained, 1:500), containing 0.1% Triton X-100, for 2 h. The samples were observed under a fluorescence microscope (Olympus FSX100) and photographed. The area of spread of platelets was calculated using NIH Image J software.

### Immunoprecipitation and western blotting

After platelets were activated, reactions were terminated by addition of an equal volume of 2X ice-cold lysis buffer (100 mM Tris/HCl pH 7.4, 1% Triton X-100, 3 mM EGTA) containing 2X protease inhibitor and 2X phosphatase inhibitor cocktail. After incubation on ice for 30 min, the samples were centrifuged at 14,800 rpm for 10 min, the supernatant was removed and incubated with the indicated antibodies in rotation at 4 °C for 2 h, followed by incubation with protein G sepharose beads for 1 h. Protein G sepharose beads were washed three times with 1X lysis buffer and SDS reducing buffer was added to the beads. The beads were boiled for 3 min. Precipitated proteins or whole cell lysate were separated by 8% SDS-PAGE and electrophoretically transferred onto a PVDF membrane. Membranes were blocked with 5% BSA in PBS. After extensive washing with Tris-buffered saline (TBS) containing 0.1% Tween 20, the membrane was incubated with antibodies at room temperature for 2 h. Antibody binding was detected using IRDye 800-conjugated goat anti-mouse IgG or IRDye 680-conjugated goat anti-rabbit IgG and visualized with the Odyssey Infrared Imaging System (LI-COR).

### Flow cytometric analysis

Washed platelets or PRP was incubated with antibodies plus PBS (negative control), poly(PHG), or convulxin, CRP, or thrombin for 15 min without stirring at room temperature, followed by fixation with 1% paraformaldehyde in PBS [32]. Samples were analyzed by performing flow cytometry using a FACScan instrument and CellQuest software (BD Biosciences).

### Murine platelets isolation and analysis of TAM receptor expression

Wild-type mice (C57BL/6 J strain) were placed under terminal anesthesia using rodent ketamine cocktail and blood was drawn into 0.3 mL of acid-citrate dextrose (ACD) solution from inferior vena cava. The blood was mixed with 100 mM EGTA containing modified Tyrode’s calcium-free buffer (EGTA buffer) and centrifuged at 180 x g at 22 °C for 10 min. The platelet-rich plasma was collected and mixed with 0.25 μM prostaglandin E1 containing EGTA buffer (washing buffer) and centrifuged at 1250 x g at 22 °C for 10 min. The pellet containing mainly platelets was collected and washed twice as above using washing buffer. The platelet pellet was resuspended and counted. TAM receptor expression on the surface was analyzed by flow cytometry using anti-mouse CD41 (133,913 (Biolegend), anti-mouse Tyro3 (FAB759P R&D), anti-mouse Axl (FAB8541P R&D), anti-mouse Mertk (12–5751-80 (eBioscience) antibodies.

### Intravital microscopy of laser-induced thrombosis of the cremaster muscle arterioles

Laser-induced injury of the mouse cremaster muscle arteriole was performed as previously described [33–35]. Alexa 488-labeled anti-CD41 F(ab)2 fragments (BD Biosciences) were infused at 0.1 μg g^− 1^ into the jugular vein. After 5 min, arterioles (30 to 45 μm diameter) were injured using a laser ablation system (Intelligent Imaging Innovations (I3)) using a Zeiss microscope (Axio Examiner D1) parfocal objective as the focal plane. The laser power was set to 55–65%, and the laser fired at the vessel wall in 1 to 3 pulses until thrombi were induced. Approximately 10 thrombi were studied in a single mouse. Injuries in which puncture of the vessel occurred or injuries in which no thrombus formed were excluded. The average number of laser pulses/injury was equal for all conditions. Data were captured using a CCD camera (Cool SnapTM HQ2) using Slidebook 5.0 image acquisition and analysis software (I3). Data were collected for 5 min after vessel wall injury. Image analysis was performed using Slidebook Version 5.0 (I3). Data were obtained from a total of 30 thrombi per group for each experimental condition. The method for the in vivo experiments was performed as described [33, 36, 37].

### Generation of recombinant proteins expressing the extracellular domain of TAM

The cDNA expressing the soluble form of the extracellular domain of TAM was subcloned into a pEF2-SPFL vector that provided a signal peptide followed by the Flag epitope fused in frame with the TAM extracellular domain. As such, these plasmids have signal sequences that allow the coded protein to be secreted from mammalian cells. Soluble TAM protein (s-TAM) was expressed by mammalian cells and purified from conditioned medium using ANTI-FLAG® M2 Affinity Gel (A2220, Sigma-Aldrich).

### Statistical analysis

Data were analyzed using the statistical software GraphPad Prism 5. For parametric comparison, the values were expressed as the mean ± SEM, one-way ANOVA (analysis of variance) followed by Tukey’s multiple comparison test or Dunnett’s multiple comparison test. The two-tailed Student’s t-test for 2 groups was used. The statistics for the in vivo experiments were performed as described [37, 38]. For the non-parametric comparison between multiple groups the area under the curve (AUC) of median FI over 300 s was analyzed with a one-way ANOVA, followed by the Kruskal-Wallis test [37, 38]. A *P* value less than 0.05 was considered significant.

## Results

### Impaired aggregation of platelets in Tyro3^−/−^ or Axl^−/−^, but not in Mertk ^−/−^ platelets in response to GPVI agonist stimulation

**Fig. 1:**
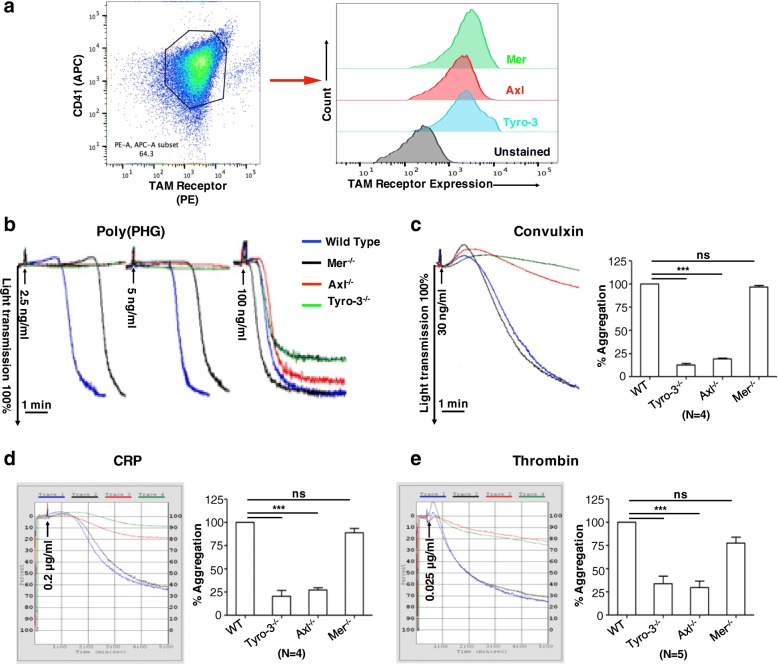
The similar expression levels of TAM receptors on the platelet surface and the decreased aggregation of Tyro3^−/−^ and Axl^−/−^ platelets, but not Mertk^−/−^ platelets. The binding of anti-mouse Mertk antibody, anti-mouse Axl antibody and anti-mouse Tyro-3 antibody to resting platelets (**a**). Washed platelets from wild type, Tyro3^−/−^, Axl^−/−^, and Mertk^−/−^ mice were stimulated with poly(PHG) at the indicated concentrations (**b**), 30 ng mL^− 1^ convulxin **(c)**, 0.2 μg mL^− 1^ CRP (**d**), or 0.025 U mL^− 1^ Thrombin (**e**). Platelet aggregation over 5 min was detected by changes in light transmission. The bar graphs represent the change in percentage of light transmission. Mean ± SEM, NS = not significant, *** *P* < 0.001. One-way ANOVA and Dunnett’s multiple comparison test

The contribution(s) of individual TAM receptors in platelet activation remains incompletely understood [23–28]. To investigate the contribution of each receptor individually, we first purified murine platelets from C57BL/6 J strain, and analyzed TAM receptor expression on the surface of platelets by flow cytometry (Fig. 1a). As indicated, murine platelets express all three TAM receptors at similar expression levels. However, despite overlapping expression, it is not clear whether they have unique or distinct functions in platelet biology and thrombosis.

To address this query, we examined single TAM receptor knockout mice individually, focusing on platelet activation mediated by three specific glycoprotein VI (GPVI) receptor agonists to compare functional outcomes in a side-by-side manner. GPVI agonists used in study are (i) poly(PHG), (ii) CRP, and (iii) convulxin. Poly(PHG) is a synthetic collagen fiber peptide for specific activation of GPVI (29), whereas CRP, Collagen-related triple helical peptide, platelet GPVI ligand [39–43]. Convulxin is a snake venom lectin that specifically activates GPVI [44, 45]. When stimulated with poly(PHG) at 2.5 and 5 ng mL^− 1^, wild-type platelets underwent full aggregation, however, platelets from Axl^−/−^ or Tyro3^−/−^ mice failed to aggregate. In contrast, Mertk^−/−^ platelets formed aggregates to the same extent as wild-type platelets but at a slower rate (Fig. 1b). The defect in the aggregation of Axl^−/−^ or Tyro3^−/−^ platelets was compromised when stimulated with high concentrations of poly(PHG), 100 ng mL^− 1^, suggesting that their phenotype results from the functional abnormality. Similarly, Axl^−/−^ or Tyro3^−/−^ platelets did not aggregate well in response to 30 ng mL^− 1^ convulxin (Fig. 1c) or CRP at 0.2 μg mL^− 1^ (Fig. 1d), although Mertk^−/−^ platelets aggregated at the same level as wild-type platelets. Platelet aggregation was restored when stimulated with a high concentration of CRP (Additional file 1: Figure S1A). Moreover, when the protease-activated receptors PAR4 were stimulated with the agonist thrombin at 0.025 U mL^− 1^, Axl^−/−^ and Tyro3^−/−^ platelets also did not aggregate well, but Mertk^−/−^ platelets aggregated similar to the wild-type platelets (Fig. 1e). The defect in Axl^−/−^ and Tyro3^−/−^ platelet aggregation was overcome when stimulated with a high concentration of thrombin (0.06 U mL^− 1^, Additional file 1: Figure S1B). Taken together, despite that all three TAMs are uniformly expressed on murine platelets, our results indicate they have distinct effect on platelets, whereby Axl and Tyro3 are critical for platelet aggregation from the above-mentioned platelet agonists that induce inside-to-outside signaling.

### Deficiency of Axl and Tyro3 receptor but not Mertk receptor inhibit integrin αIIbβ3 activation

**Fig. 2:**
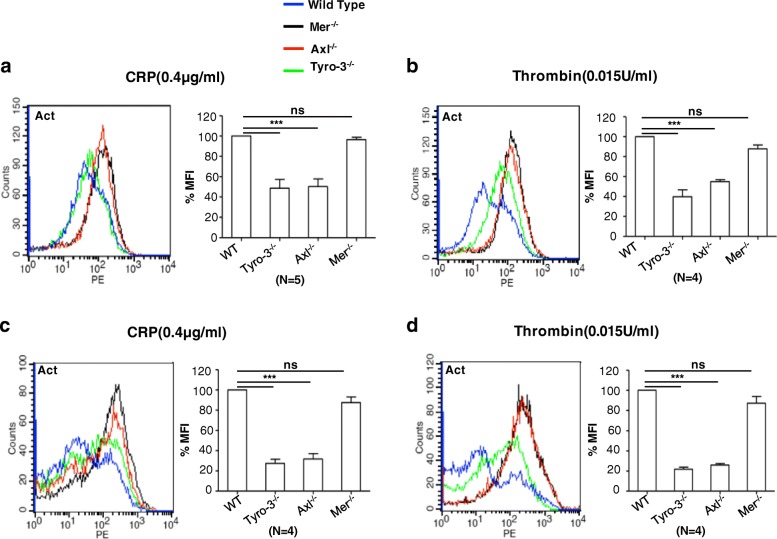
The decreased JON/A binding and P-selectin expression in Tyro3^−/−^, Axl^−/−^ but not Mertk^−/−^ platelets. Washed platelets from wild type, Tyro3^−/−^, Axl^−/−^ or Mertk^−/−^ mice were stimulated with 0.4 μg mL^− 1^ CRP or 0.015 U mL^− 1^ thrombin, followed by incubation with PE-labeled JON/A antibody (**a**, **b**) and PE-labeled anti-P-selectin antibody (**c**, **d**). The samples were analyzed using flow cytometry. MFI = mean fluorescent intensity. Mean ± SEM, NS = not significant, *** *P* < 0.001. One-way ANOVA and Dunnett’s multiple comparison test

The suppressed platelet aggregation resulting from a deficiency of Tyro3 and Axl receptors suggests that these receptors may also affect the activation of the integrin αIIbβ3. To test this hypothesis, washed platelets were stimulated with 0.4 μg mL^− 1^ CRP (Fig. 2a) or 0.015 U mL^− 1^ thrombin (Fig. 2b) and activation of the αIIbβ3 was assessed by binding of PE-labeled JON/A, which specifically recognizes activated mouse integrin αIIbβ3. Similar to the platelet aggregation shown in Fig. 1, JON/A binding on wild-type and Mertk^−/−^ platelets was largely increased, but not on Axl^−/−^ or Tyro3^−/−^ platelets. When platelet-rich plasma (PRP) was stimulated with 50 ng mL^− 1^ poly(PHG), JON/A binding to platelets was similar among wild-type and TAM-knockout platelets (Additional file 1: Figure S2 Ai), while stimulation with the higher concentration of poly(PHG),1 μg mL^− 1^, binding of JON/A to the platelets was observed similar to the CRP and thrombin (Additional file 1: Figure S2 Aii-Aiii). Without activation, JON/A binding on wild-type, Mertk^−/−^, Axl^−/−^, and Tyro3^−/−^ platelets were similar (Additional file 1: Figure S2 Bi). In addition, stimulation with 3.5 μg mL^− 1^ convulxin (Additional file 1: Figure S2 Bii- Biii), significantly increased JON/A binding on wild-type and Mertk^−/−^ platelets, but not on Axl^−/−^ or Tyro3^−/−^ platelets was observed. Analogous to the results described above, these data suggest that Axl and Tyro3 receptor are required for integrin αIIbβ3 activation.

### Secretion of α-granule is impaired in Tyro3^−/−^ and Axl^−/−^ platelets

Aggregation-dependent platelet α-granule secretion is essential for the formation of stable macroaggregates after initial formation of small and reversible platelet aggregates. To test whether TAM receptors are involved in platelet secretion, we measured the membrane expression of P-selectin, a marker of platelet α-granule release, by flow cytometry. Washed platelets were stimulated either with 0.4 μg mL^− 1^ CRP (Fig. 2c) or 0.015 U mL^− 1^ thrombin (Fig. 2d) and P-selectin expression was analyzed. The P-selectin expression was higher on wild-type or Mertk^−/−^ platelets, but not on Axl^−/−^ or Tyro3^−/−^ platelets. When using the PRP and stimulating platelets with 0.5 μg mL^− 1^ poly(PHG), increased expression was P-selectin expression was observed on wild-type and TAM knockout platelets (Additional file 1: Figure S3 Ai). Stimulation at higher concentration of poly(PHG), 2 μg mL^− 1^(Additional file 1: Figure S3 Aii-Aiii), or 2.5 μg mL^− 1^ convulxin (Additional file 1: Figure S3 Bi) increased P-selectin expression on wild-type and Mertk^−/−^ platelets, but not on Axl^−/−^ or Tyro3^−/−^ platelets was observed. Without activation with agonists, P-selectin expression on wild-type, Mertk^−/−^, Axl^−/−^, and Tyro3^−/−^ platelets was similar. These findings suggest that Axl and Tyro-3, but not Mertk, plays a role in the α-granule secretion.

### Deficiency of Tyro3 and Axl, but not Mertk, inhibit platelet spreading

**Fig. 3:**
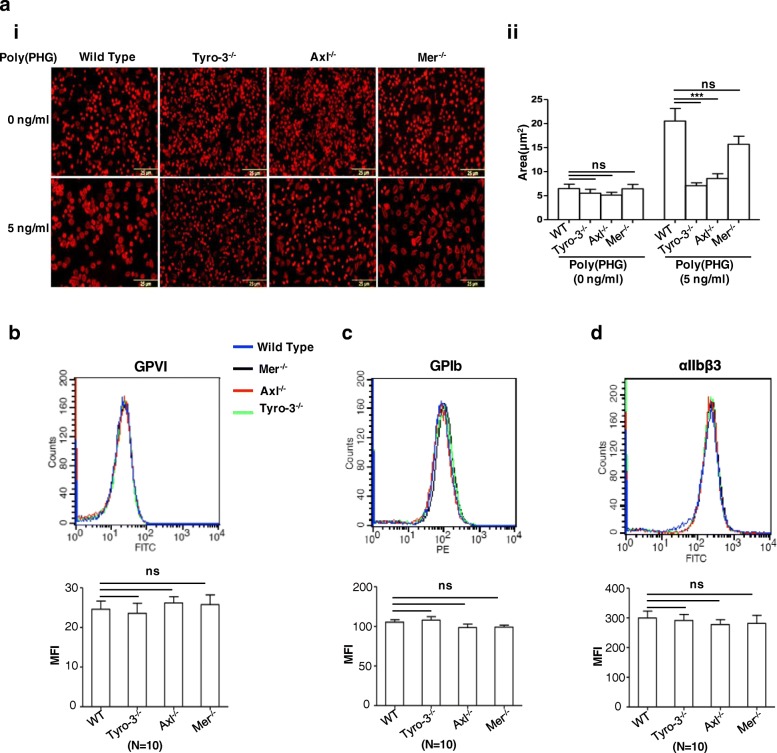
The defect of spreading of Tyro3^−/−^ and Axl^−/−^ platelets stimulated with poly(PHG) on a fibrinogen-coated surface. Washed platelets from wild-type, Tyro3^−/−^, Axl^−/−^, or Mertk^−/−^ mice were seeded on coverslips coated with 10 μg mL^− 1^ fibrinogen in the presence or absence of 5 ng mL^− 1^ poly(PHG) for 30 min. After washing, adherent platelets were fixed in 4% paraformaldehyde at room temperature for 30 min and stained with TRITC-conjugated phalloidin (1:500) containing 0.1% Triton X-100 for 2 h. The samples were observed under a fluorescent microscope and photographed using the Olympus FSX100. Representative images are shown (**a** i) and histograms represent mean ± SEM of five independent experiments (**a** ii). Expression levels of glycoproteins (αIIbβ3, GPIb, or GPVI) on platelets from wild-type, Tyro3^−/−^, Axl^−/−^, and Mertk^−/−^ mice was analyzed by flow cytometry (**b**-**d**). MFI = mean fluorescent intensity. NS = not significant, *** P < 0.001. One-way ANOVA and Dunnett’s multiple comparison test

We further investigated role of TAM receptors on the platelet activation by analyzing platelet spreading. Mouse platelets were incubated on fibrinogen-coated coverslips and stimulated with 5 ng mL^− 1^ poly(PHG). Five ng mL^− 1^ poly(PHG) significantly stimulated lamellipodia formation in the wild-type and Mertk^−/−^ platelets, but not in Tyro3^−/−^ and Axl^−/−^ platelets (Fig. 3a). In the presence of poly(PHG), the area of wild-type platelets undergoing spreading was 20.523 ± 2.641 μm^2^, which was significantly larger than that of Tyro3^−/−^ platelets (6.016 ± 0.513 μm^2^) and Axl^−/−^ platelets (8.573 ± 1.012 μm^2^), but it was similar to that of Mertk^−/−^ platelets (15.707 ± 1.673 μm^2^) (Fig. 3aii). We further examined whether the defect in platelet activation resulted from the down-regulation of membrane receptors or integrin αIIbβ3 levels in the absence of TAM receptors by analyzing the surface expression of GPVI, GPIb, and integrin αIIbβ3 on the platelets, however their expression levels were comparable among wild-type and the TAM single knockout platelets (Fig. 3b-d). Thus, the loss of TAM receptors does not affect the expression of functional glycoproteins on the platelet membrane but ablation of Axl and Tyro3 receptors significantly affects the platelet spreading.

### Axl and Tyro-3, but not Mertk are required for platelet accumulation

**Fig. 4:**
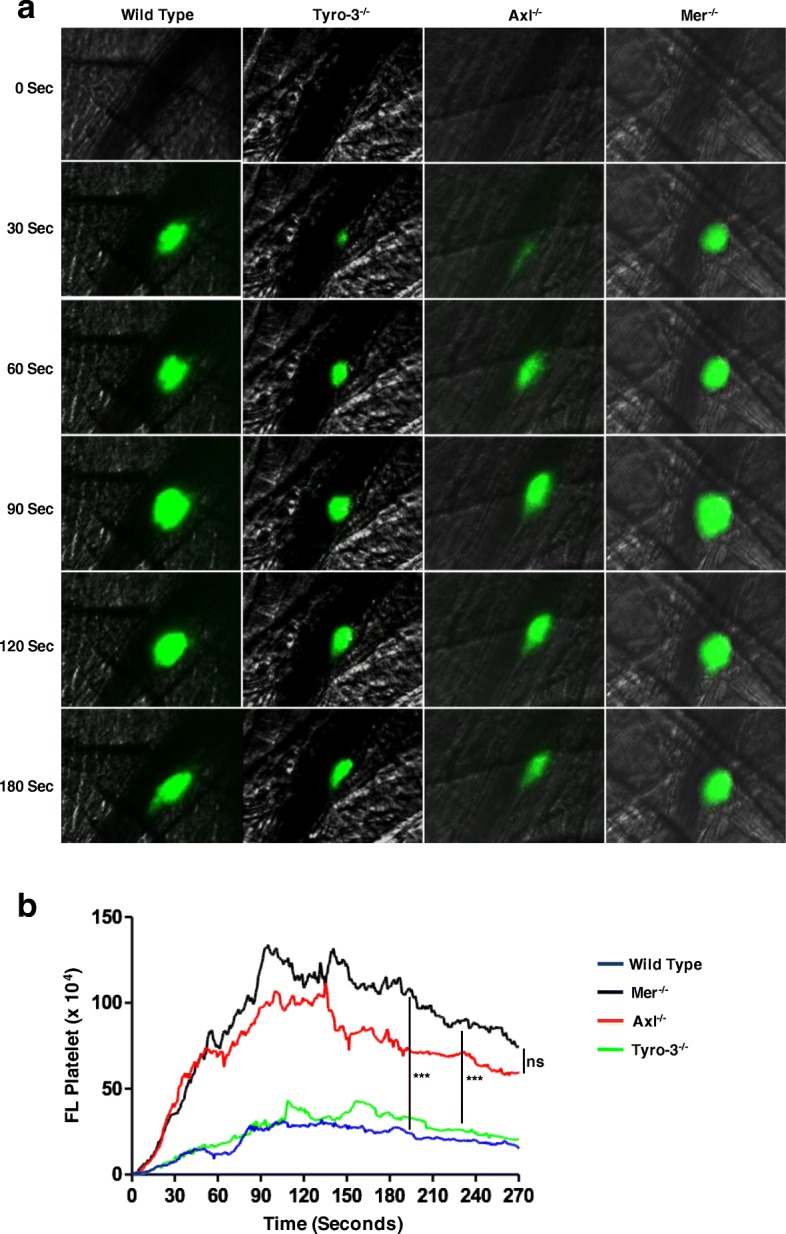
Tyro3 and Axl, but not Mertk, are required for platelet accumulation in vivo. Cremaster arteriole injury was induced in Tyro3^−/−^, Axl^−/−^, Mertk^−/−^, and wild-type mice. Platelets were detected using anti-CD41 F(ab)2 fragments (binding to platelet αIIb) conjugated with Alexa Fluor 488. (**a**) Using intravital microscopy, representative fluorescence images show platelet accumulation (green) at selected time points up to 180 s after vascular injury. The median integrated fluorescence intensities (FI) of anti-CD41 antibody over 270 s (platelet**, b**). The data were analyzed by the area under the curve (AUC) with one-way ANOVA and Kruskal-Wallis test. The data were obtained from 30 thrombi in 3 mice for each experimental condition. NS = not significant, *** P < 0.001

To confirm whether the role of TAM receptors in platelet activation in vitro is consistent with that of platelet accumulation in vivo, we have used a laser-induced injury model as described previously [33, 36, 37]. Thrombosis was analyzed with fluorescence intravital microscopy. Following laser-induced cremaster arteriolar wall injury, platelet accumulation was visualized by infusion of Alexa Fluor 488-conjugated anti-CD41 Fab fragments. The role of TAM receptor in the platelet accumulation was examined using wild-type, Mertk^−/−^, Axl^−/−^, and Tyro3^−/−^ mice. The median integrated fluorescence intensity obtained from quantitative data from multiple thrombi indicated that Axl^−/−^ and Tyro3^−/−^, but not Mertk^−/−^ mice have defects in platelet accumulation at the sites of injury as compared to control, wild-type mice (Fig. 4a,b). These data suggests that Axl and Tyro-3, but not Mertk receptor is required for platelet accumulation in vivo.

### Stimulated protein tyrosine phosphorylation is suppressed in platelets lacking the Tyro3 or Axl receptor

**Fig. 5:**
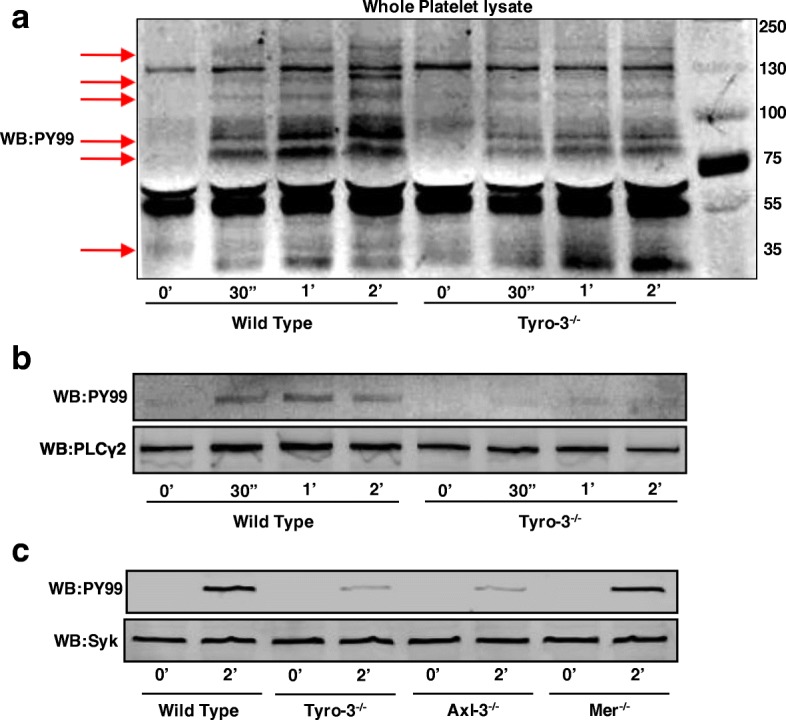
GPVI-stimulated protein tyrosine phosphorylation is inhibited in the absence of Tyro3 and Axl, but not Mertk. After wild-type and Tyro3^−/−^ platelets were stimulated with 30 ng mL^− 1^ convulxin for the indicated time period, platelets were solubilized and the lysates were subjected to immunoprecipitation with antibody against PLCγ2. Whole cell lysates (**a**) or anti-PLCγ2 immunoprecipitates (**b**) were analyzed for tyrosine phosphorylation by immunoblotting using PY99. Equal protein loading was assured by probing the samples with antibody against PLCγ2. (**c**) Tyro3^−/−^, Axl^−/−^, Mertk^−/−^, and wild-type platelets were stimulated with 30 ng mL^− 1^ convulxin for 2 min, platelets were solubilized and the lysates were subjected to immunoprecipitation with antibody against Syk. The anti-Syk immunoprecipitates were analyzed for tyrosine phosphorylation using PY99. The blots are representative of three independent experiments

The results above suggest that Tyro3 and Axl receptors participate in platelet activation and thrombosis. Therefore, to further test whether intracellular signaling during platelet activation is regulated by TAM receptors, we investigated the GPVI signaling pathway. As shown in Fig. 5a, convulxin stimulates tyrosine phosphorylation of a series of proteins in wild-type platelets in a time-dependent manner. Interestingly, tyrosine phosphorylation of proteins with molecular weights between 70 and 150 kDa was attenuated in Tyro3^−/−^ platelets. It is well known that GPVI elicits a signal transduction cascade that involves sequential activation of Syk, LAT, and SLP-76, leading to PLCγ2 activation [41, 46–51]. To determine whether the deficiency of Tyro3 inhibits this pathway, PLCγ2 was immunoprecipitated and its tyrosine phosphorylation was evaluated. The tyrosine phosphorylation of PLCγ2 was strongly induced in wild-type platelets stimulated with convulxin, while it was completely suppressed in Tyro3^−/−^ platelets. Similar phosphorylation of PLCγ2 was also obtained for Axl^−/−^, but not for Mertk^−/−^ platelets (data not shown). In order to further understand the regulation of TAM receptors on the upstream signaling molecule PLCγ2, we collected wild-type and TAM single knockout platelets stimulated with convulxin at two time points, zero and two minutes, and determined tyrosine phosphorylation of Syk using immunoprecipitation. As shown in Fig. 5c, tyrosine phosphorylation of Syk was strongly suppressed in Tyro3^−/−^ and Axl^−/−^ platelets, but not in Mertk^−/−^ platelets. The above findings imply that the Tyro3 and Axl receptors are important for the inside-to-outside signaling involving PLCγ2 and GPVI signaling.

### Trans-interaction of Axl or Tyro3 may mediate contact-dependent activation

**Fig. 6:**
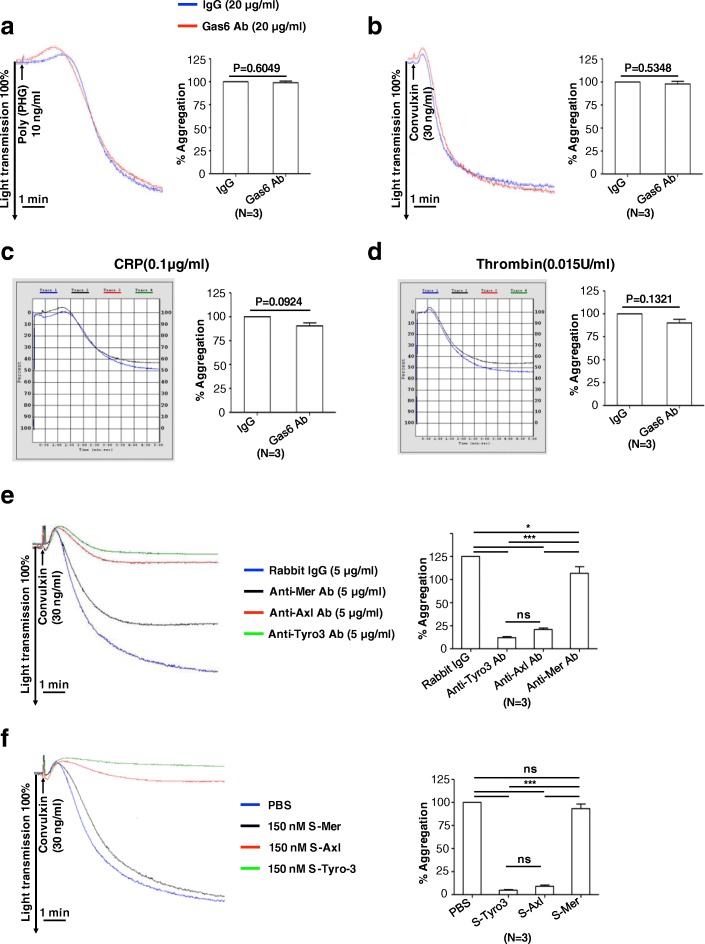
The trans-interaction of Axl or Tyro3, but not receptor ligation is not involved in platelet activation. After incubation with 20 μg mL^− 1^ anti-Gas6 antibody or isotype control IgG for 5 min, human platelets at a density of 2 × 10^8^ mL^− 1^ were stimulated with 10 ng mL^− 1^ poly(PHG) (**a**), 30 ng mL^− 1^ convulxin (**b**), 0.1 μg mL^− 1^ CRP (**c**), or 0.015 U mL^− 1^ thrombin (**d**) to induce aggregation. Mean ± SEM, NS = not significant. *n* = 3, Student’s t-test. After human platelets were preincubated with 5 μg mL^− 1^ control IgG, anti-Tyro3 Ab, anti-Axl Ab, and anti-Mertk Ab, respectively for 5 min, they were stimulated with 30 ng mL^− 1^ convulxin to induce aggregation(**e**). After preincubation with 150 nM recombinant extracellular domain of Axl, Tyro3, and Mertk for 5 min, human platelets were stimulated with 30 ng mL^− 1^ convulxin (**f**). Mean ± SEM, n = 3, NS = not significant, *P < 0.05, *** P < 0.001. One-way ANOVA and Tukey’s multiple comparison test

The above-mentioned results indicate that Tyro3 and Axl, but not Mertk, participate in an inside-out GPVI to TAM signaling mechanism in platelets, leading to collagen -mediated platelet aggregation and subsequently thrombus formation. In other cell types, TAM receptors have been shown to mediate ligand-independent cell-cell contact formation via homophilic interactions of extracellular domains [52, 53]. Hence, to further investigate whether TAMs also participate in outside-in signaling and/or heterotypic trans-interactions that depend on their extracellular domains, we tested a series of extracellular blocking agents that included (i) anti-Gas6 neutralizing antibody (Fig. 6a-d) (ii) anti-TAM neutralizing mAbs (Fig. 6e) and (iii) TAM ectodomain soluble receptor traps (Fig. 6f), to investigate whether autocrine-inside to outside signaling also contributes in this model.

**Fig. 7:**
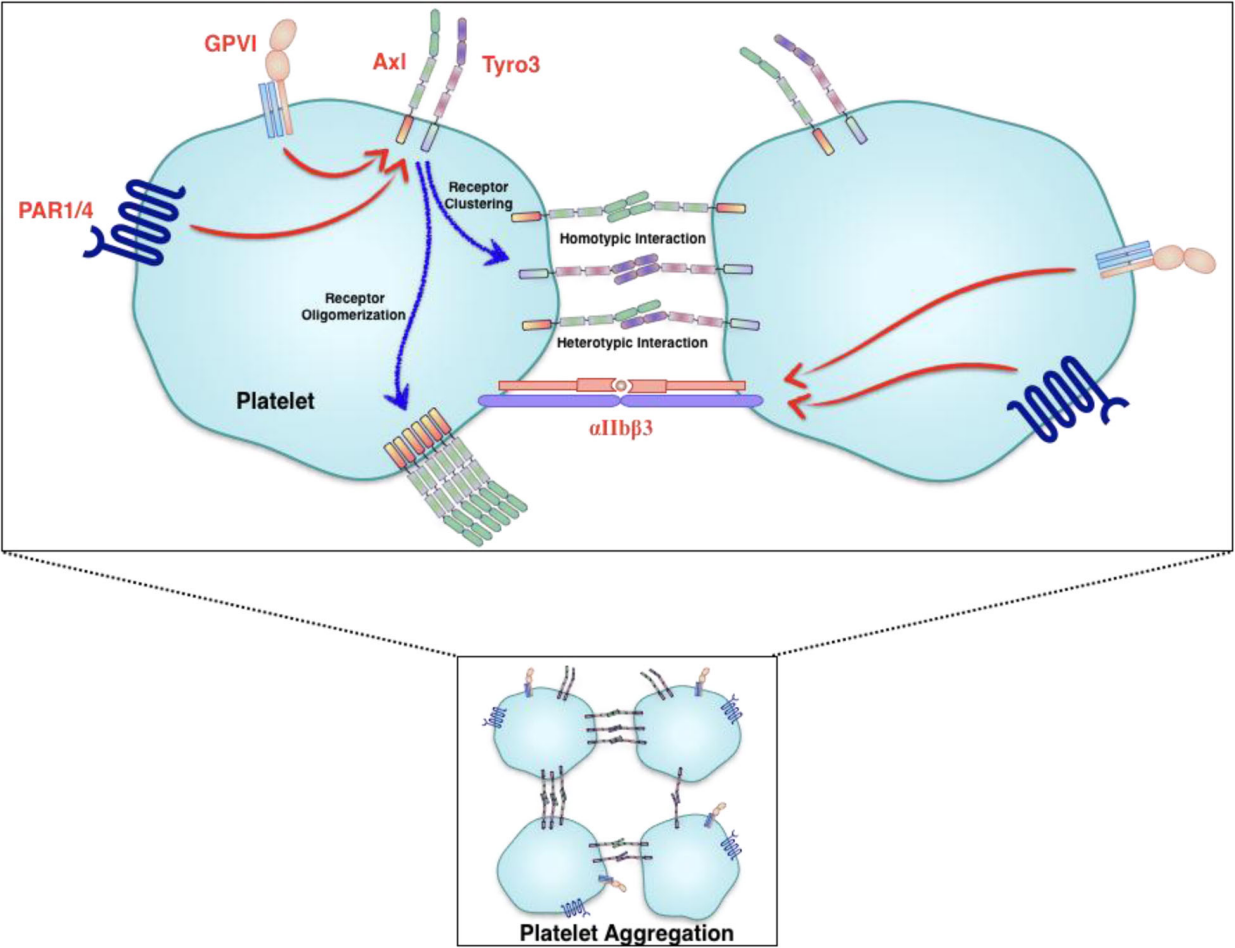
Schematic representation of the role of TAM receptors in platelet activation. Trans-interaction of Axl or Tyro3 might mediate contact-dependent activation, facilitating integrin αIIbβ3 activation signaling

To analyze autocrine-inside to outside signaling, we pre-treated murine platelets with either IgG control or Gas6 Ab (20 μg/ml) following stimulation with several GPVI agonists, poly(PHG) (10 ng/ml) (Fig. 6a), convulxin (30 ng/ml) (Fig. 6b), CRP (0.1 μg/ml) (Fig. 6c), or thrombin (0.015 U/ml) (Fig. 6d). As noted, under these conditions, we failed to see blockage of platelet aggregation under these conditions. The results suggest that autocrine stimulation of TAMs, via locally autocrine produced Gas6 may not be responsible for the observed inside-to-outside GPVI mediated TAM-dependent platelet activation. To more formally test this idea, we treated platelets with either anti-TAM mAbs to the extracellular domains (Fig. 6e) or ectodomain soluble traps for TAM receptor (Fig. 6f) following stimulation with convulxin (30 ng/ml). The pre-incubation with an anti-Tyro3 or an anti-Axl antibody strongly inhibited convulxin induced platelet aggregation. However no effect of anti-Mertk antibody was observed on the platelet aggregation (Fig. 6e). Notably, these antibodies recognize the extracellular portion of the receptors. Additionally, pretreatment with soluble recombinant proteins against extracellular domains of Axl and Tyro3, but not that of Mertk, inhibited convulxin-stimulated platelet aggregation (Fig. 6f). Taken together, these data suggest that trans-interaction of Axl or Tyro3 can mediate contact-dependent activation, facilitating the activation of signaling and integrin αIIbβ3 activation leading to the platelet activation and aggregation (Fig. 7).

## Discussion

Formation of early thrombi after vascular injury begins with the local exposure of collagen and generation of thrombin, followed by the tethering of circulating platelets by collagen and the subsequent accumulation of aggregated platelets. Once activated, platelets form persistent contact with each other, allowing molecules on the surface of adjacent platelets to interact *in trans* and facilitate thrombus growth and stability. Thus, understanding the spectrum of molecules involved and their activation mechanisms are important. This information is expected to reveal the processes of initial thrombus formation and help in identifying novel targets for anti-thrombotic therapy.

Previously, in other cell types, the trans-interaction of TAM receptors is one example of contact-dependent signaling [52, 53]. In this study, we provided evidence that the TAM receptors Axl and Tyro3 have an important function in platelet activation and thrombosis, including aggregation, integrin αIIbβ3 activation, α-granule release, platelet spreading, and platelet accumulation in vivo (Fig. 1-3). In contrast, Mertk appears to be dispensable for platelet activation under the conditions used in this study. Contrary to previous studies showing that TAM receptors are equally important in platelet activation mediated by physiologic agonists such as ADP and thrombin [24], our results demonstrate that platelet activation and thrombosis mainly rely on Axl and Tyro3. Loss of Axl or Tyro3 expression causes a defect in platelet activation and thrombosis, demonstrating that Axl and Tyro3 have unique roles in signaling pathway required for platelet activation (Fig. 4).

Currently, the mechanisms by which Tyro3 and Axl participate in GPVI and thrombin-mediated PAR signaling is not completely understood, although this likely depends on inside to outside signaling mechanism and trans-activation of the extracellular domains. Whether this requires extracellular Gas6, or Gas6 pre-bound to Tyro3 or Axl (or Pros1 bound to Tyro3) is not resolved in this study, although we did not see an inhibitory effect on platelet aggregation using anti-Gas6 antibody that binds to the receptor-binding region. A previous study has shown that plasma Gas6 levels do not influence platelet aggregation [54], so it is conceivable that the Axl/Tyro3-mediated effects for platelet activation and thrombosis are ligand- independent. It is known for example, that in other cell types, TAM receptors may function as cell adhesion receptors in a ligand-independent manner [52, 53, 55]. Indeed, the tandem Ig/FN type III domain structure of TAM extracellular domains are arranged similarly to cell adhesion molecules, such as intercellular adhesion molecules and vascular cell adhesion molecules [56–58]. Moreover, at the molecular level, the Ig domains of Tyro3 form dimers in vitro, both in the crystal and in solution [53], and when Tyro3 is overexpressed either at the cell surface or in the cytoplasm, it can form dimers even in the absence of its ligand [55]. A similar mode of action for Axl was also proposed [52].

Accordingly, in the aforementioned model, in order to allow cell adhesion to occur, TAM receptors displayed on the surfaces of opposing cells may form dimers through homophilic interactions. Although the contribution of a single homophilic interaction might be expected to be weak, a large cluster of dimerized receptors would be sufficient to promote stable platelet-platelet contact at the initial step of receptor stimulation and is probably a prerequisite for full platelet activation. As noted above, this model has been presented for a number of cell adhesion molecules of the Ig superfamily [57, 58] (Fig. 7). The fact that the anti-Tyro3 antibody and the anti-Axl antibody strongly inhibit platelet aggregation (Fig. 6e), as well as their soluble ectodomains (Fig. 6f) that competitively binds to their counterpart receptors in order to inhibit aggregation support the idea that trans-interaction of Axl and Tyro3 is important for platelet activation. We envision that during the initial response to collagen, platelets may utilize Axl and Tyro3 to form stable and close contact with each other, thereby amplifying stimulation of receptors on platelets such as GPVI and PAR1/4. Since both Axl-deficient and Tyro3-deficient platelets have a defective phenotype, Axl and Tyro3 may constitute a unique amplification system in normal platelet function and their roles are not redundant.

In addition to the homotypic or heterotyopic interactions within the extracellular domains of Tyro3 and Axl that may mediate aggregation, it is also possible that GPVI and/or PAR receptors induce inside to outside signaling in order to cluster Tyro3 or Axl into higher order aggregates. For example, we have previously shown that Tyro3 (and Mertk) can become hyper-activated in the presence of phosphatidylserine liposomes or apoptotic cells/vesicles, a signaling event predicted to be mediated by receptor clustering [59, 60]. Additional studies, using mutagenesis in the Ig and FN type III domains, or cryo-EM could be used to better address queries about trans-interactions and receptor clustering. Likewise. it will also be equally important to understand the cause for Mertk being apparently less important in platelet activation and thrombosis, given that Mertk is also sufficiently expressed on platelets. Whether Mertk plays more of a role in outside to inside signaling in platelets, or other signaling role, should be further investigated. Future studies addressing these issues will help in the understanding of the mechanism of platelet activation and thrombosis.

In this study, we provide evidence showing that trans-interaction of Axl or Tyro3 mediate contact-dependent activation, thereby facilitating the activation of platelets and thrombosis. The present results lead to a revised concept for the role of TAM receptors in platelet activation and thrombosis positing that Axl or Tyro3 mediate trans-interaction and contact-dependent activation, thereby facilitating activation of signaling, α-granule release, and integrin αIIbβ3 activation during aggregation. Identification of the specific functions of Axl and Tyro3 should reveal their potential as therapeutic targets for anti-thrombotic treatment.

## Conclusions

Axl and Tyro-3, but not Mertk, are required to support platelet activation and aggregation. Tyro3 and Axl, but not Mertk receptor is required for thrombus thrombosis. Axl and Tyro-3 mediate the phosphorylation of Syk for platelet function. TAM receptors regulate platelet activation independently of ligand binding. Trans-interaction of Axl or Tyro3 might mediate contact-dependent activation, thereby facilitating the activation signaling and integrin αIIbβ3 activation.

## Additional file

Additional file 1: **Figure S1**. The defect in aggregation of TAM single knockout platelets were compromised when stimulated by high concentration of agonists. (A) CRP(0.6mg mL-1)-induced aggregation (n=4) and (B) Thrombin (0.06U mL-1)-induced aggregation (n=4); mean ±SEM*,* NS = not significant. One-way ANOVA followed by dunnett's multiple comparison test. **Figure S2.** JON/A binding is decreased on Tyro3-/-and Axl-/-platelets on in response to poly (PHG) and convulxin. Platelets from wild type, Tyro3-/-, Axl-/-or Mer-/-mice were stimulated with poly(PHG) **(**Ai-Aiii) and convulxin(Bi-Biii), followed by incubation with PE-labeled JON/A antibody. The samples were analyzed by flow cytometry. Mean ±SEM, n=3, NS = not significant, ***P<0.001. One-way ANOVA followed by dunnett's multiple comparison test. **Figure S3**. The deficiency of Tyro3 and Axl inhibits P-selectin expression on platelet surface in response to poly (PHG) and convulxin. Platelets from wild type, Tyro3-/-, Axl-/- or Mer-/- mice were stimulated with poly(PHG) (Ai-Aiii) and convulxin(Bi-Biii), followed by incubation with PE-labeled anti-P-selectin antibody. The samples were analyzed by flow cytometry. Mean ±SEM, n=3, NS = not significant, ***P<0.001. One-way ANOVA followed by dunnett's multiple comparison test. (PDF 290 kb)

## Abbreviations

CRP: Collagen-related triple-helical peptide
Gas6: Growth arrest-specific gene 6
GPVI: Glycoprotein VI
PAR1/4: Protease-activated receptors PAR1 and PAR4
PLCγ2: Phospholipase Cγ2
rhGas6: Recombinant human Gas6 protein
Syk: Spleen tyrosine kinase
TAM: Receptor tyrosine kinases, Tyro3, Axl and Mertk
