# Supplementary material for: Tyro3, Axl, and Mertk receptors differentially participate in platelet activation and thrombus formation
Source: Cell Commun Signal. 2018 Dec 12;16:98. doi: 10.1186/s12964-018-0308-0 (PMC6291976; doi:10.1186/s12964-018-0308-0)
Supplement: Supplementary file 1 — Figure S1. The defect in aggregation of TAM single knockout platelets were compromised when stimulated by high concentration of agonists. (A) CRP(0.6mg mL-1)-induced aggregation (n=4) and (B) Thrombin (0.06U mL-1)-induced aggregation (n=4); mean ±SEM, NS = not significant. One-way ANOVA followed by dunnett's multiple comparison test. Figure S2. JON/A binding is decreased on Tyro3-/-and Axl-/-platelets on in response to poly (PHG) and convulxin. Platelets from wild type, Tyro3-/-, Axl-/-or Mer-/-mice were stimulated with poly(PHG) (Ai-Aiii) and convulxin(Bi-Biii), followed by incubation with PE-labeled JON/A antibody. The samples were analyzed by flow cytometry. Mean ±SEM, n=3, NS = not significant, ***P<0.001. One-way ANOVA followed by dunnett's multiple comparison test. Figure S3. The deficiency of Tyro3 and Axl inhibits P-selectin expression on platelet surface in response to poly (PHG) and convulxin. Platelets from wild type, Tyro3-/-, Axl-/- or Mer-/- mice were stimulated with poly(PHG) (Ai-Aiii) and convulxin(Bi-Biii), followed by incubation with PE-labeled anti-P-selectin antibody. The samples were analyzed by flow cytometry. Mean ±SEM, n=3, NS = not significant, ***P<0.001. One-way ANOVA followed by dunnett's multiple comparison test. (PDF 290 kb) [file 12964_2018_308_MOESM1_ESM.pdf]

Supplemental figure I.

A CRP

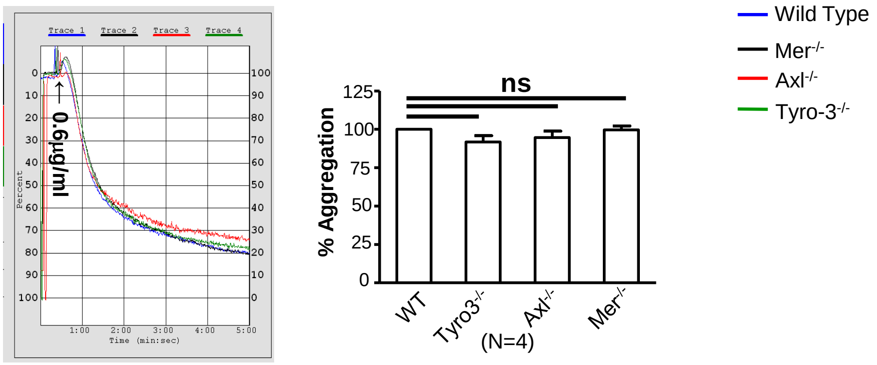

B Thrombin

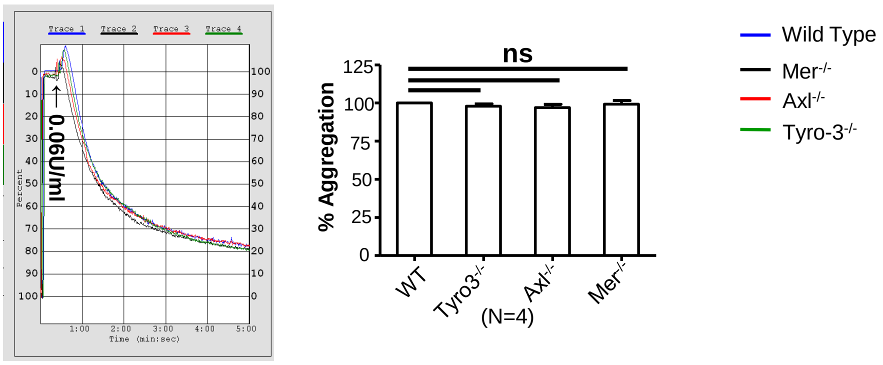

Supplemental figure I. The defect in aggregation of TAM single knockout platelets were compromised when stimulated by high concentration of agonists. **(A)** CRP(0.6μg mL^{-1})-induced aggregation(n=4) and **(B)** Thrombin(0.06U mL^{-1})-induced aggregation(n=4); mean ± SEM, NS = not significant. One-way ANOVA followed by dunnett's multiple comparison test.

Supplemental figure II.

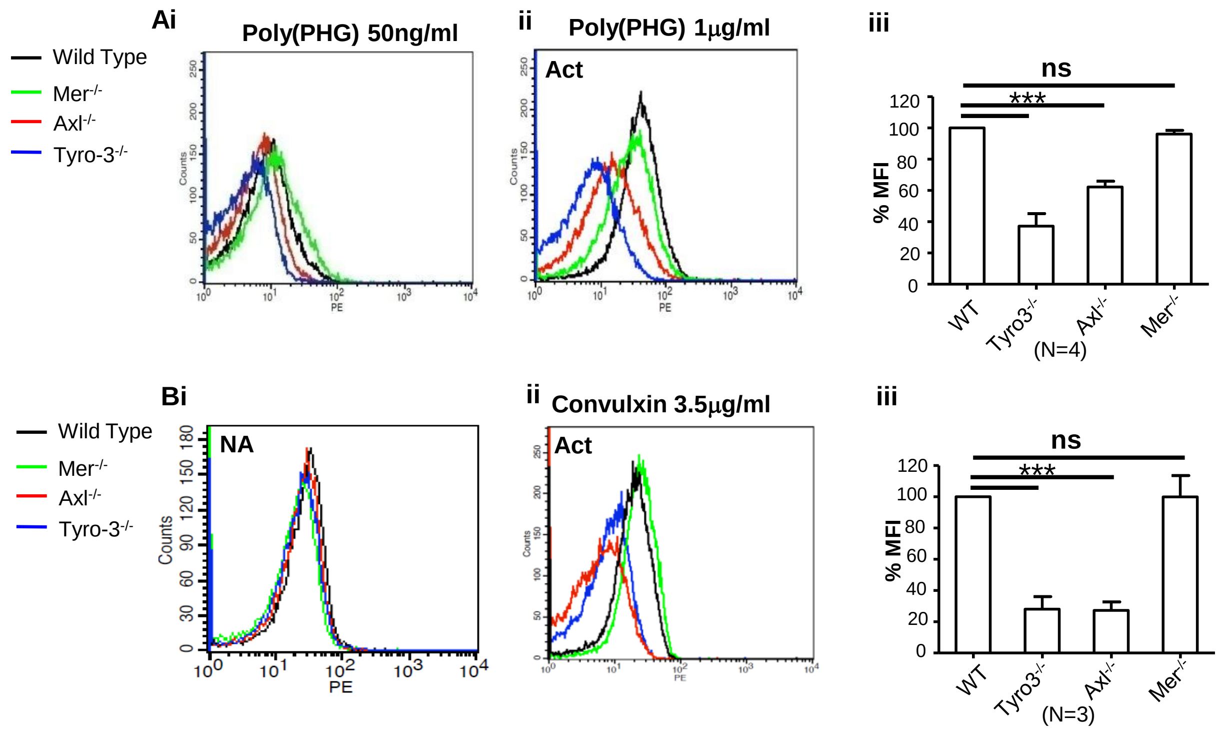

Supplemental figure II. JON/A binding is decreased on Tyro3$^{-/-}$ and Axl$^{-/-}$ platelets on in response to poly(PHG) and convulxin. Platelets from wild type, Tyro3$^{-/-}$, Axl$^{-/-}$ or Mer$^{-/-}$ mice were stimulated with poly(PHG) **(Ai-Aiii)** and convulxin **(Bi-Biii),** followed by incubation with PE-labeled JON/A antibody. The samples were analyzed by flow cytometry. Mean ± SEM, n=3, NS = not significant, *** P<0.001. One-way ANOVA followed by dunnett's multiple comparison test.

Supplemental figure III.

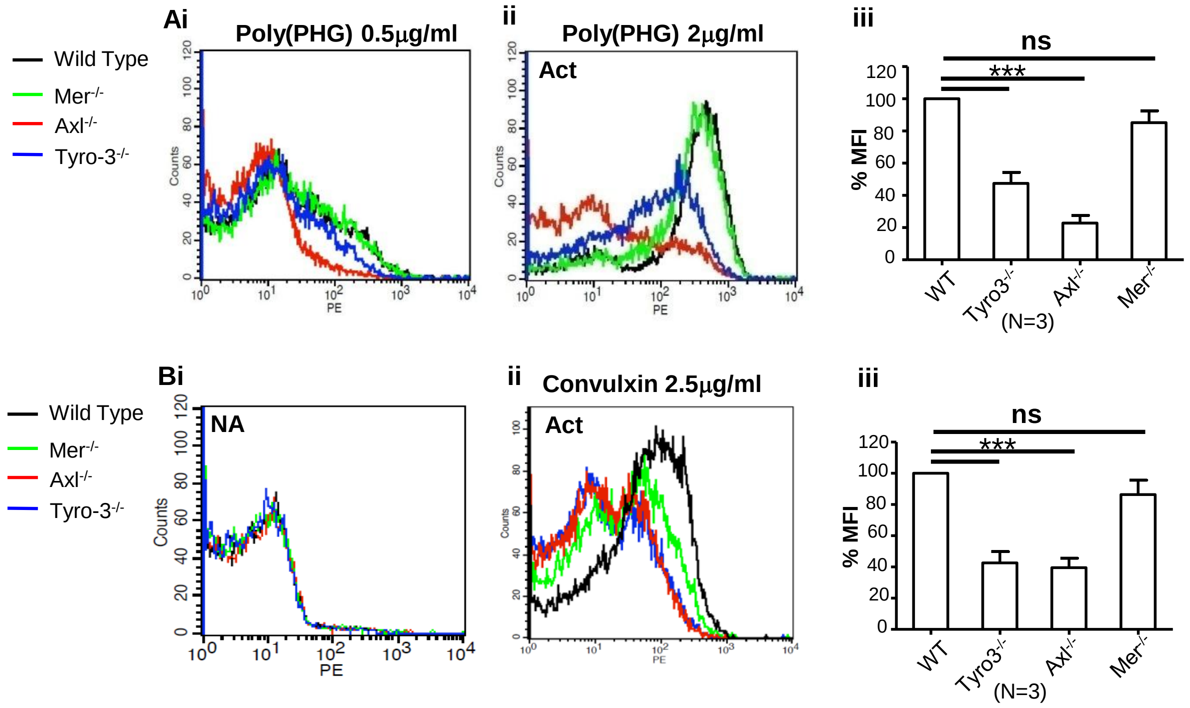

<u>Supplemental figure III</u>. The deficiency of Tyro3 and Axl inhibits P-selectin expression on platelet surface in response to poly(PHG) and convulxin. Platelets from wild type, Tyro3$^{-/-}$, Axl$^{-/-}$ or Mer$^{-/-}$ mice were stimulated with poly(PHG) **<u>(Ai-Aiii)</u>** and **<u>convulxin(Bi-Biii),</u>** followed by incubation with PE-labeled anti-P-selectin antibody. The samples were analyzed by flow cytometry. Mean ± SEM, n=3, NS = not significant, *** P<0.001. One-way ANOVA followed by dunnett's multiple comparison test.
